# Ab-normal saline in abnormal kidney function: risks and alternatives

**DOI:** 10.1007/s00467-018-4008-1

**Published:** 2018-07-09

**Authors:** Wesley Hayes

**Affiliations:** 1 Great Ormond Street Hospital, London, UK; 2 University College London Institute of Child Health, London, UK

**Keywords:** Metabolic acidosis, Saline solution, Water-electrolyte balance, Acute kidney injury, Chronic kidney disease

## Abstract

Intravenous 0.9% saline has saved countless lives since it was introduced over a century ago. It remains the most widespread crystalloid in both adult and pediatric practice. However, in recent years, evidence of deleterious effects is accruing. These include increased mortality, acute kidney injury (AKI), metabolic acidosis, and coagulopathy. The predominant cause for these sequelae appears to be the excess chloride concentration of 0.9% saline relative to plasma. This has led to development of balanced isotonic solutions such as PlasmaLyte. This review summarizes current evidence for adverse effects of chloride-rich intravenous fluid and considers whether 0.9% saline should still be used in 2018 or abandoned as a historical treatment in favor of balanced crystalloid solutions.

## Introduction

Administration of intravenous fluid directly affects patients’ extracellular fluid and electrolyte status. While these effects can be therapeutic, some are unintended, and have the potential to cause harm. The most commonly prescribed intravenous crystalloid in pediatric practice is currently 0.9% saline. There is accruing evidence for adverse effects, most recently in a large randomized trial demonstrating increased mortality in critically ill adults [1]. The predominant cause for these sequelae appears to be the excess chloride concentration of 0.9% saline relative to plasma. Patients with abnormal kidney function have reduced capacity to excrete excess chloride, which increases their risk of these complications.

In recent years, balanced intravenous solutions with physiological chloride concentration have been developed as isotonic alternatives to 0.9% saline. This review outlines current evidence for the effects of excess chloride in 0.9% saline and considers whether its use in clinical practice is still justified.

## Chloride in intravenous fluid

Pediatric practice has changed over the last decade with a shift away from hypotonic intravenous fluids such as 0.45% saline or 0.2% saline to predominantly 0.9% saline. The rationale for this change is avoidance of iatrogenic hyponatremia. Many children in hospital are at increased risk of hyponatremia due to vasopressin release stimulated by nausea, pain, gastroenteritis, lung pathology, or the postoperative state [2, 3]. Hyponatremia is of particular concern because it predisposes to intracellular fluid shifts and brain edema which can cause permanent neurological impairment or even death [4]. Rapid correction of hyponatremia can also result in neurological complications such as osmotic demyelination. Systematic reviews have highlighted the risk of hyponatremia from hypotonic intravenous fluid [5–8]. For the majority of children, isotonic intravenous fluids mitigate these risks [9], although specific disorders such as diabetes insipidus mandate hypotonic solutions because isotonic fluids lead to hypernatremia and cerebral myelinosis [10].

The most commonly prescribed intravenous fluid is currently 0.9% saline which was developed at the turn of the twentieth century [11]. While the tonicity and sodium concentration of 0.9% saline are within 10% of physiological levels, its chloride concentration exceeds that of plasma by approximately 60% (Table 1). In contrast, balanced intravenous fluids have chloride concentrations closely aligned to that of plasma. Traditional balanced solutions are Hartmann’s or Ringer’s lactate [12]. Solutions with even closer alignment to plasma constituents have subsequently been developed such as PlasmaLyte (Table 1).

**Table 1:** Intravenous fluid constituents and properties (with plasma ranges given in first row)

Fluid	Osmolality (mOsm/l)	Na^+^(mmol/l)	K^+^(mmol/l)	Mg_2+_(mmol/l)	Cl^−^(mmol/l)	Acetate (mmol/l)	Gluconate (mmol/l)	Lactate (mmol/l)
Plasma	275–295	135–145	3.5–5.3	0.8–1.2	95–108	(Bicarbonate 20–32)		
PlasmaLyte 148	295	140	5	1.5	98	27	23	
Hartmann’s	278	131	5		111			29
0.9% saline	308	154			154			
0.45% saline 4% glucose	376	77			77			
0.18% saline 4% glucose	284	31			31			

There is accruing evidence for deleterious effects resulting from excess chloride in patients receiving intravenous 0.9% saline. Children with impaired kidney function are at particular risk of these sequelae due to reduced capacity to excrete excess chloride. The potential mechanisms underlying the effects of chloride-rich crystalloid will now be discussed.

## Effects of chloride

### Chloride and mortality

A growing body of evidence highlights an increased mortality risk in association with administration of chloride-rich intravenous fluid.

In a preclinical model of sepsis, fluid resuscitation with 0.9% saline was associated with more metabolic acidosis and inferior survival than balanced resuscitation fluid [13].

The association of chloride-rich intravenous fluid and mortality is increasingly recognized in critically ill adults. In an analysis of 53,448 adults with sepsis and acute kidney injury (AKI), patients who received greater proportions of chloride-rich fluid experienced increased mortality [14]. A recent cluster-randomized, multiple crossover trial in 15,802 adults on intensive care found a higher rate of a composite outcome of death, new renal replacement therapy, or persistent renal dysfunction in patients who received 0.9% saline as opposed to balanced intravenous crystalloid [1].

In keeping with adult studies, pediatric evidence for a link between excess chloride and mortality is emerging. In an analysis of 890 children with septic shock in 29 pediatric intensive care units in the USA, hyperchloremia was independently associated with inferior outcomes including mortality and complicated clinical course [15]. This was corroborated in a further single-center study of 66 children on pediatric intensive care [16].

Notwithstanding the association of chloride-rich intravenous fluid and hyperchloremia with increased mortality, hypochloremia is also associated with adverse outcomes. Low serum chloride levels were a strong predictor of mortality in a 10-year follow-up study of over 9000 adults [17]. The physiology underlying this association remains to be elucidated, but the effect appears to be independent of plasma sodium or potassium concentrations [18, 19].

The mechanism underlying increased mortality in patients who receive large volumes of chloride-rich crystalloid has not been definitively established; however hyperchloremic metabolic acidosis is strongly implicated. The physiological basis for this phenomenon will be outlined in the next section.

### Chloride and acid-base balance

Hyperchloremic metabolic acidosis is consistently observed in both adult and pediatric patients following fluid resuscitation with 0.9% saline [20, 21]. A Cochrane systematic review of postoperative intravenous fluid management in adult patients concluded that chloride-rich fluid, as opposed to balanced fluid, significantly increases the risk of metabolic acidosis [22].

The cause of metabolic acidosis following an intravenous chloride load will now be discussed.

#### Etiology of saline-induced hyperchloremic metabolic acidosis

There are two schools of thought for understanding the mechanisms of acute acid-base disturbances. In the traditional Henderson-Hasselbalch and Siggaard-Anderson line of thinking, acute alterations in plasma pH are explained by changes in partial pressure of carbon dioxide (pCO_2_) and thereby carbonic acid [23]:

The Henderson-Hasselbalch equation

$$ \mathrm{pH}=\mathrm{pK}+\log\ \left({\mathrm{HCO}}_{3^{-}}/{\mathrm{pCO}}_2\times 0.225\right) $$

Using the Henderson-Hasselbalch approach, the link between excess chloride from 0.9% saline and metabolic acidosis is not immediately obvious.

Stewart subsequently published an alternative approach to understanding acute acid base disturbances [24, 25]. Stewart’s strong ion theory gives a physiological rationale for excess chloride driving metabolic acidosis. It refers to principles of electro-neutrality (all negatively charged and positively charged ions must balance) and conservation of mass (the total amount of a substance remains constant, unless added to, generated, removed or destroyed). Stewart proposed that acute acid-base balance is determined by carbon dioxide, weak acids, and the strong ion difference. Strong ions are almost completely ionized in solution, and the plasma strong ion difference is defined as

$$ \mathrm{Strong}\ \mathrm{ion}\ \mathrm{difference}=\left(\left[{\mathrm{Na}}^{+}\right]+\left[{\mathrm{K}}^{+}\right]+\left[{\mathrm{Ca}}^{2+}\right]+\left[{\mathrm{Mg}}^{2+}\right]\right)\hbox{--} \left(\left[{\mathrm{Cl}}^{-}\right]+\left[\mathrm{lactate}\right]\right) $$

Stewart proposed that changes in the strong ion difference directly alter plasma pH. If a large volume of intravenous 0.9% saline is administered, this will reduce the plasma strong ion difference via a higher relative increase in plasma [Cl^−^] than [Na^+^]. This reduction in strong ion difference will increase dissociation of water to H^+^ and OH^−^, thereby driving metabolic acidosis.

**Fig. 1:**
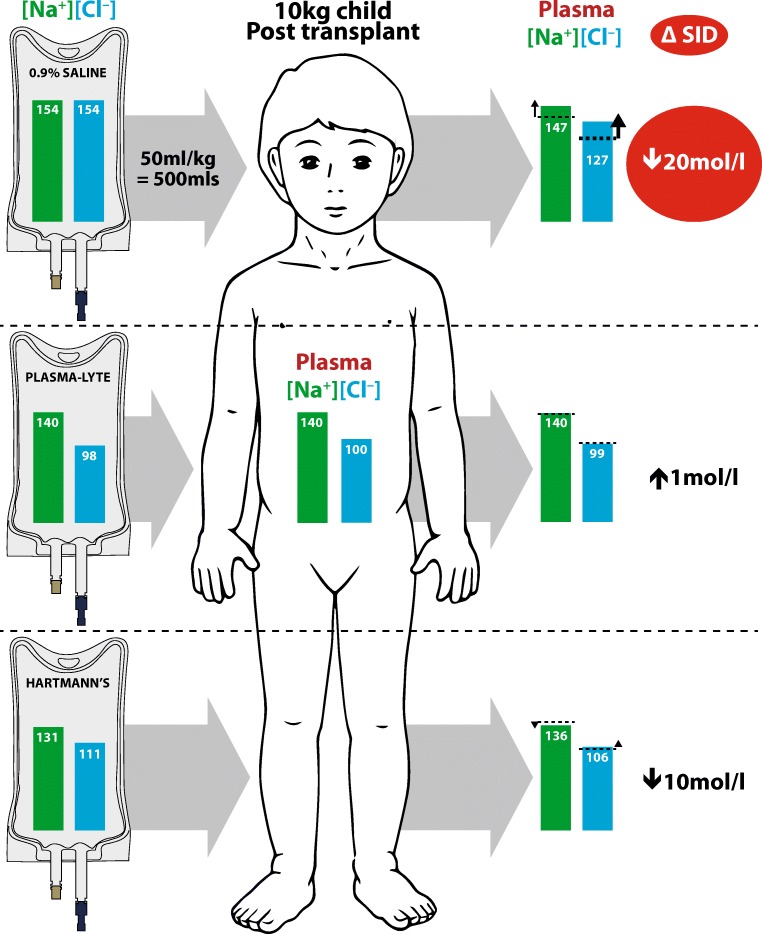
Effects of administering 50 ml/kg of three different intravenous fluids to an anuric child postkidney transplant with hematocrit 37.5% and plasma volume 500 ml (ΔSID change in strong ion difference)

To illustrate this phenomenon, the effect of 50 ml/kg intravenous crystalloid (0.9% saline, PlasmaLyte and Hartmann’s) on the plasma strong ion difference in a 10 kg child who is anuric postkidney transplant is shown in Fig. 1. A significant change in strong ion difference results from 0.9% saline which thereby causes metabolic acidosis. The effect of Hartmann’s solution on plasma strong ion difference is smaller, and that of PlasmaLyte is negligible, thereby having no significant effect on the patient’s acid base balance (Fig. 1).

Alternative explanations for saline-induced metabolic acidosis have also been proposed. They include dilutional acidosis, which suggests that infusion of buffer-free crystalloid dilutes the bicarbonate concentration in extracellular fluid thereby precipitating acidosis. However, a nonlinear relationship between extracellular volume expansion and reduction in bicarbonate concentration does not support this concept [26, 27].

Stewart’s strong ion theory of acute acid-base disturbance provides a physiologically plausible rationale for the consistent clinical observation that infusion of chloride-rich fluid results in metabolic acidosis. Given that metabolic acidosis affects myocardial function, renal and intestinal perfusion, nerve function, and extracellular potassium buffering, it is likely to play a role in the increased mortality observed in critically ill patients who receive large volumes of 0.9% saline.

### Chloride and kidney perfusion

Data from preclinical studies and clinical trials suggest a direct effect of chloride on intra-renal perfusion, vasoconstriction, and glomerular filtration.

In animal studies, infusion of chloride-rich fluid in denervated kidneys resulted in vasoconstriction and reduction in glomerular filtration rate [28]. In healthy adult volunteers, renal artery flow velocity and renal cortical tissue perfusion fell significantly from baseline after infusion of 2 litres 0.9% saline, but not after PlasmaLyte [29]. A chloride-liberal intravenous fluid strategy was associated with more AKI and renal replacement therapy than a chloride-restrictive strategy in a study of 760 adult patients on intensive care [30]. A recent multiple-crossover trial comparing 0.9% saline with balanced crystalloids in 13,347 noncritically ill adults attending an emergency department found a higher incidence of major adverse kidney events following 0.9% saline [31]. These findings were not corroborated in the SPLIT trial, which compared incidence of AKI in adults receiving 0.9% saline with PlasmaLyte; however, the power of this study was limited by small volumes of fluid infused in patients with moderate severity of disease [32]. In summary, although no pediatric studies have been published to date, data from pre-clinical models and adult patients strongly implicate chloride-rich intravenous fluid in acutely reducing kidney blood flow and function.

### Chloride and coagulation

There is a consistent theme from preclinical models and clinical studies in adults undergoing major surgery that chloride-rich resuscitation fluid impairs coagulation.

In an animal model of hemorrhagic shock, resuscitation with 0.9% saline was associated with hyperchloremic acidosis and coagulopathy that were not observed with lactated Ringer’s solution [33]. In adult patients undergoing major abdominal surgery, chloride-rich intravenous fluid was associated with increased blood product requirements [34, 35].

The anticoagulant effect of 0.9% saline has been ascribed to dilutional coagulopathy related to the significantly larger volume of saline required for resuscitation relative to balanced fluids [33]. Further work is needed to definitively delineate the underlying pathophysiology; nevertheless, these data constitute a compelling case for balanced intravenous fluid in surgical care.

### Chloride and fluid overload

Intravenous fluid can be highly effective in restoring tissue perfusion in patients with intravascular depletion [36]. Conversely, injudicious use can result in fluid overload. In children with normal kidney function, fluid overload is uncommon because feedback mechanisms adjusting urine volume and natriuresis are intact [37]. However, in children with AKI or chronic kidney disease (CKD), extracellular fluid volume homeostasis is often perturbed.

Studies in adult patients suggest that chloride-rich crystalloid may increase the risk of fluid overload to a greater degree than balanced solutions. In a study of healthy adult volunteers, urinary excretion of water and sodium were inferior following infusion of 2 litres 0.9% saline when compared to Hartmann’s solution [38]. In the surgical setting, patients receiving 0.9% saline for volume resuscitation required more blood products than those who received balanced crystalloids [34, 35]. Blood products increase the risk of circulatory overload and its sequelae [39]. Taken together, these data suggest an increased risk of fluid overload in patients receiving 0.9% saline as opposed to balanced solutions.

Fluid overload has serious sequelae. It is associated with increased mortality in critically ill children and neonates with AKI [40–43]. It is a key contributor to cardiovascular morbidity and mortality in children with CKD [44]. Avoiding fluid overload is therefore a key priority, and the potential role of excess chloride merits further evaluation.

### Chloride and blood pressure

While the effect of salt on intravascular volume is well known, there is accruing evidence to suggest that chloride may influence blood pressure in its own right [45]. Outside the hospital setting, the majority of dietary chloride is consumed as NaCl, so the distinction between sodium and chloride is academic; however, differentiating the individual contribution of these ions to blood pressure homeostasis may be important when selecting intravenous fluid.

Several lines of evidence implicate chloride in systemic hypertension. Animal and clinical studies found that the hypertensive effect of enteral sodium chloride was not replicated with sodium citrate or phosphate [46–48]. Further, preclinical data suggest a specific effect of chloride [49] and other halides [50] on blood pressure and renal vascular resistance. Recent understanding of hypertonic electrolyte sequestration in the skin, and the role of macrophages in regulation of this storage, has led to the observation that skin chloride accumulation is associated with systemic hypertension. [51]

With-no-lysine kinases (WNKs) are serine threonine kinases which play a major role in the regulation of sodium and potassium transport in the distal nephron [52] and in monogenic hypertension [53]. The importance of intracellular chloride in WNK signaling was recently elucidated in a Drosophila model: reduction in intracellular chloride in combination with the regulatory protein Mo25 were found to stimulate WNK activity and transcellular ion flux in the renal epithelium [54]. This may represent one of the mechanisms by which chloride affects blood pressure.

While further studies are needed, the importance of chloride in blood pressure regulation is increasingly recognized.

## The case for abandoning 0.9% saline

Given the accruing evidence for deleterious effects of excess chloride, should physicians abandon chloride-rich crystalloid, which was developed over a century ago, in favor of isotonic balanced solutions such as PlasmaLyte? A number of studies in adult patients and some pediatric data support this argument.

Traditionally, 0.9% saline was the resuscitation fluid of choice to manage intravascular depletion. The following data suggest that this is no longer appropriate: When used as resuscitation fluid in children on intensive care, balanced fluids were associated with lower mortality, lower prevalence of AKI, and lower inotrope requirements in a propensity-matched analysis of over 3000 children from 43 centers [55]. Similarly, adult studies demonstrate a consistent link between chloride-rich resuscitation fluid and subsequent mortality in critically ill patients [14, 56]. Observational data were recently corroborated in a rigorously conducted randomized study in over 15,802 adults which confirmed that balanced crystalloids result in a lower rate of a composite outcome of death from any cause, new renal-replacement therapy, or persistent renal dysfunction than 0.9% saline [1].

Further studies evidence inferior surgical outcomes in patients receiving 0.9% saline. In an observational analysis of over 30,000 adult patients undergoing abdominal surgery, postoperative use of 0.9% saline was associated with metabolic acidosis, increased mortality, increased infection rates, and increased blood product requirement, compared to PlasmaLyte [35].

Inferior outcomes are consistently reported in patients at risk of AKI who receive 0.9% saline. Increased incidence of AKI was found in a study of 760 adults when a chloride-liberal intravenous fluid strategy was used, as opposed to a chloride-restrictive strategy [30]. These findings were not corroborated in the randomized SPLIT trial; however, relatively small volumes of intravenous fluid were used potentially blunting a true effect [32]. A meta-analysis found an increase in AKI and mechanical ventilation time in patients in whom chloride rich resuscitation fluid was used [57].

Kidney transplantation is a further scenario in which 0.9% saline may impact outcome. Kidney transplant recipients often produce large volumes of urine due to acute tubular injury and are at increased risk of electrolyte/acid base disturbances. A Cochrane systematic review of studies in adult kidney transplant recipients concluded that perioperative normal saline was associated with more metabolic acidosis than balanced solutions [58]. A study comparing 0.9% saline to Ringer’s lactate in adult kidney transplant recipients was terminated early by the monitoring board due to significantly more hyperchloremic hyperkalemic metabolic acidosis in the saline group [59]. The finding of significantly more hyperkalemia with 0.9% saline than lactated Ringers (containing 4 mmol/l potassium) was corroborated in two further studies in adult kidney transplant recipients [60, 61]. PlasmaLyte (containing 5 mmol/l potassium) has also been shown to reduce the risk of hyperkalemia relative to potassium-free 0.9% saline in adults with end-stage kidney disease undergoing transplantation [62, 63]. Although no pediatric data are available, these findings are noteworthy because pediatric transplant recipients receive large volumes of intravenous crystalloid to maintain allograft perfusion perioperatively [64].

Although it may seem counterintuitive that potassium containing balanced crystalloids *reduce* the risk of hyperkalemia relative to potassium-free saline in patients with impaired kidney function, this is expected when the physiology of potassium homeostasis is considered. Potassium is a predominantly intracellular ion, and the small proportion of extracellular (plasma) potassium is regulated by cellular shifts in the short term. Hyperchloremic acidosis from 0.9% saline impairs cellular buffering of potassium, thereby predisposing to hyperkalemia. In contrast, the balanced electrolyte composition, together with plasma bicarbonate resulting from gluconate and acetate metabolism in patients receiving PlasmaLyte, both serve to maintain cellular potassium buffering. The relatively small quantity of potassium in balanced solutions is needed to mitigate the risk of hypokalemia. Given the physiology of potassium homeostasis, and the consistent observation that balanced solutions reduce the risk of hyperkalemia, concerns about hyperkalemia from potassium in balanced solutions are not justified.

Taken together, there is now overwhelming evidence that large volumes of 0.9% saline increase the risk of mortality, metabolic acidosis, and AKI in critically ill adults, and some data suggesting increased postoperative and transplant complications including hyperkalemia. Pediatric data are scant so definitive conclusions cannot be drawn; however, for volume resuscitation and kidney transplant care, the case for abandoning 0.9% saline in favor of balanced solutions is compelling.

## The case for keeping 0.9% saline

There are some clinical scenarios in which chloride-rich crystalloid will remain the intravenous fluid of choice. For children with hypochloremic metabolic alkalosis and intravascular volume depletion, the electrolyte composition of 0.9% saline can aid restoration of physiological electrolyte and acid-base balance.

Salt losing tubulopathies such as Bartter syndrome (prevalence 1:1,000,000) are disorders of urinary chloride wasting, with resultant hypochloremic metabolic alkalosis. Children with these conditions therefore have a high chloride requirement. While enteral supplementation with a combination of sodium and potassium chloride is preferable, intravenous fluid is required in some situations. For these patients, 0.9% saline is preferable to a balanced solution. This group is exceptional in that further addition of chloride in the form of potassium chloride, resulting in intravenous fluid with chloride concentrations up to 194 mmol/l can be indicated.

Cystic fibrosis is a further disorder which predisposes to excessive chloride loss; excessive sweating can lead to hypochloremic metabolic alkalosis [65]. While chloride-rich fluid is not universally indicated in patients with cystic fibrosis, in the context of intravascular depletion and hypochloremic alkalosis, 0.9% saline is an appropriate choice of crystalloid.

Children who experience severe persistent vomiting can develop hypochloremic alkalosis as a result of gastric hydrochloric acid loss. Infants with pyloric stenosis are at particular risk [66]. For patients with hypochloremic alkalosis due to gastric losses, 0.9% saline with additional potassium chloride can aid normalization of plasma electrolyte and acid-base balance.

For children with traumatic brain injury, the hypertonicity of 0.9% saline may mitigate the risk of cerebral edema more effectively than balanced isotonic fluids; however, there is currently no evidence to support either approach in adults or children [67].

In summary, in clinical conditions which predispose to hypochloremic alkalosis and intravascular volume depletion, 0.9% saline is preferable to balanced solutions which may not adequately compensate chloride loss. It can therefore be argued that 0.9% saline should never be completely abandoned, because it is the most appropriate crystalloid for some children as part of an individualized fluid prescription.

## Individualized intravenous fluid prescriptions

All children who require intravenous fluid need an individualized prescription that is based on their medical condition and tailored in response to changes in their fluid, electrolyte, and acid-base status. This is particularly important in children with abnormal kidney function, whether AKI or CKD. AKI is common in critically ill children and associated with increased mortality [68]. “Maintenance fluid” can be a dangerous concept for these patients in whom homeostatic mechanisms to regulate fluid, electrolytes, and acid-base are disturbed.

Special situations should be borne in mind. Children with diabetes insipidus need hypotonic fluid, and isotonic solutions are harmful. Those with traumatic brain injury may require hypertonic solutions. Children with hypochloremic alkalosis secondary to chloride loss require chloride-rich fluid, often with added potassium chloride. Such fluid should be avoided in most patients with AKI or CKD.

While the initial fluid prescription can be based on the presenting plasma chemistry and acid-base status, careful monitoring of patients’ fluid status, plasma, and urine electrolytes is paramount in order to tailor the prescription appropriately. “Standard” intravenous fluid prescriptions are rarely appropriate; choice of intravenous crystalloid should be guided by patients’ individual clinical status and adjusted in response to monitoring.

## Summary

Evidence is accruing that balanced intravenous fluids mitigate the deleterious effects of excess chloride in 0.9% saline in some clinical settings. In adult patients, balanced fluids are associated with lower mortality, less AKI, and less perioperative complications than 0.9% saline. Concerns about the potassium content of balanced fluids in patients with abnormal kidney function are not justified, since hyperchloremic acidosis from 0.9% saline confers a greater risk of hyperkalemia. Although pediatric data are scant, balanced isotonic fluids such as PlasmaLyte are probably better suited to the majority of children, particularly those with abnormal kidney function and reduced capacity to excrete excess chloride. Balanced fluids may have particular advantages when used as isotonic resuscitation fluid and in kidney transplant recipients. However, a “one-size-fits all” approach to intravenous fluid must be avoided. Prescriptions must be tailored to each individual child’s needs considering their underlying medical condition, plasma chemistry, and fluid status. Many hospitalized children do not “need” intravenous fluid at all; the enteral route is safer and should always be the default.

Multiple-choice questions (answers are provided following the reference list)

1. Hyperchloremic metabolic acidosis can be explained by:
  - An increase in the plasma strong ion difference
  - A reduction in the plasma strong ion difference
  - An increase in the plasma partial pressure of CO2
  - Inhibition of dissociation of water to ionized constituents
2. 0.9% saline has the following properties, relative to plasma:

3. PlasmaLyte contains the following constituents:
  - Na, Cl, lactate
  - Na, Cl, K, Ca, acetate, gluconate
  - Na, Cl, K, Mg, acetate, gluconate
  - Na, Cl, K, Ca, Mg, acetate
4. Deleterious effects of 0.9% resuscitation fluid in adults include:
  - Increased mortality
  - Acidosis
  - Increased blood product requirement
  - All of the above
5. Addition of 20 mmol potassium chloride to 500 ml 0.9% saline results in a chloride concentration of:
  - 154 mmol/l
  - 164 mmol/l
  - 174 mmol/l
  - 194 mmol/l
